# Establishment, Immortalisation and Characterisation of Pteropid Bat Cell Lines

**DOI:** 10.1371/journal.pone.0008266

**Published:** 2009-12-11

**Authors:** Gary Crameri, Shawn Todd, Samantha Grimley, Jennifer A. McEachern, Glenn A. Marsh, Craig Smith, Mary Tachedjian, Carol De Jong, Elena R. Virtue, Meng Yu, Dieter Bulach, Jun-Ping Liu, Wojtek P. Michalski, Deborah Middleton, Hume E. Field, Lin-Fa Wang

**Affiliations:** 1 CSIRO Livestock Industries, Australian Animal Health Laboratory, Geelong, Australia; 2 Australian Biosecurity Cooperative Research Centre for Emerging Infectious Diseases, Brisbane, Australia; 3 Monash Immunology and Stem Cell Laboratories, Monash University, Melbourne, Australia; 4 Queensland Primary Industries and Fisheries, Biosecurity Queensland, Brisbane, Australia; 5 Department of Microbiology and Immunology, The University of Melbourne, Melbourne, Australia; 6 Central Clinical School, Monash University, Melbourne, Australia; Charité-Universitätsmedizin Berlin, Germany

## Abstract

**Background:**

Bats are the suspected natural reservoir hosts for a number of new and emerging zoonotic viruses including Nipah virus, Hendra virus, severe acute respiratory syndrome coronavirus and Ebola virus. Since the discovery of SARS-like coronaviruses in Chinese horseshoe bats, attempts to isolate a SL-CoV from bats have failed and attempts to isolate other bat-borne viruses in various mammalian cell lines have been similarly unsuccessful. New stable bat cell lines are needed to help with these investigations and as tools to assist in the study of bat immunology and virus-host interactions.

**Methodology/Findings:**

Black flying foxes (*Pteropus alecto*) were captured from the wild and transported live to the laboratory for primary cell culture preparation using a variety of different methods and culture media. Primary cells were successfully cultured from 20 different organs. Cell immortalisation can occur spontaneously, however we used a retroviral system to immortalise cells via the transfer and stable production of the Simian virus 40 Large T antigen and the human telomerase reverse transcriptase protein. Initial infection experiments with both cloned and uncloned cell lines using Hendra and Nipah viruses demonstrated varying degrees of infection efficiency between the different cell lines, although it was possible to infect cells in all tissue types.

**Conclusions/Significance:**

The approaches developed and optimised in this study should be applicable to bats of other species. We are in the process of generating further cell lines from a number of different bat species using the methodology established in this study.

## Introduction

There is increasing evidence to indicate that bats play a major role in the emergence and transmission of new and deadly zoonotic viruses [1]. Bats are the putative natural reservoir hosts for a number of emerging zoonotic viruses including Nipah virus (NiV) [2], Hendra virus (HeV) [3], severe acute respiratory syndrome coronavirus (SARS-CoV) [4], [5] and Ebola virus [6]. These agents are among some of the most virulent pathogens to emerge from animal reservoirs and are capable of infecting a broad range of species. The discovery of SARS-like coronaviruses (SL-CoVs) in Chinese horseshoe bats [4], [5] has triggered attempts internationally to isolate a SL-CoV from a variety of bat species. However, this has so far been unsuccessful and attempts to isolate other bat viruses in various mammalian cell lines have been equally difficult. The two bat cell lines available commercially, Tb1-Lu (ATCC number CCL-88, derived from the lung of *Tadarida brasiliensis*) and Mvi/It (ATCC number CRL-6012, established from a interscapular tumour of *Myotis velifer incautus*), are of limited value for comprehensive studies since they are not susceptible to infection with viruses of interest (Crameri, G., unpublished results). A greater variety of bat cell lines from a wide range of tissue types is urgently needed for in-depth studies.

Bats are genetically diverse, highly mobile and are dispersed across every continent except Antarctica [1]. They are taxonomically classified into the order Chiroptera, with two suborders, the Megachiroptera (megabats) and the Microchiroptera (microbats). Many bat species exhibit a high infection tolerance towards harboured pathogens, making bats a favourable host of different viruses and also a critical target for medical and veterinary research. The Chiroptera have remained evolutionarily unchanged for over 35 million years [7] and so it might be expected that bat viruses would have developed a sophisticated interaction with the host immune system as a result of extensive co-evolution over a long period of time.

Although many of the pathogens that bats carry are capable of inducing severe systemic illness in diverse terrestrial mammalian hosts, they are comparatively innocuous in bats [2], [6]. Under experimental conditions, it has been shown that infection of bats, with a range of viruses is largely subclinical, with low levels of viral genome detectable in tissues, viral shedding at the limits of detection, and inconsistent or transient seroconversion [8], [9]. Research into bat biology, immunology in particular, and bat-virus interaction will provide valuable insights into the mechanisms of infection and pathogenesis, and may lead to novel approaches to manage and prevent bat virus disease outbreaks affecting animals and humans. As infection studies in wild caught bats are difficult and pose risks to natural populations of bats, the development of new stable bat cell lines for *in vitro* studies are essential and would greatly reduce the dependence on the use of live bats.

One of the major issues facing the establishment of stable primary cell culture is natural cell cycle death, which normally occurs after a pre-programmed number of cell divisions[10]. There are several strategies which can be employed to immortalise cell lines. The first involves the introduction and stable expression of genes coding for the Simian virus 40 large T and small t tumour antigens (SV40T). The large T antigen acts by binding to and attenuating tumour suppressor protein p53 and the proteins of the retinoblastoma tumour suppressor family (pRb, p130 and p107).These changes alter the cell cycle to promote DNA replication and cell division [11], [12], [13]. Intracellular expression of the gene coding for the SV40 large T antigen is a well established, directed recombinant approach to the production of immortalised cell lines [14], [15] and has been used to immortalise cells from a number of species including human [16], rabbit [17] and rat [18].

The second approach to cell immortalisation relies on the introduction and stable expression of the catalytic subunit of the human telomerase reverse transcriptase (hTERT). In the absence of hTERT, telomeres are shortened with repeated cell divisions resulting in cells entering a state of senescence then cell death, inferring that telomere length is a possible factor in the determination of the replicative life span of human cells [19]. The ectopic expression of hTERT has been successfully used to immortalise primary cell lines in a range of mammalian species including goat mammary epithelial cells [20], bovine microvascular endothelial cells [21], canine Schwann cells [22], swine kidney epithelial cells [23] and human myometrial [24], retinal pigment epithelial cells and foreskin fibroblasts [25]. In most cases, unlike SV40T immortalisation, this approach results in minimal phenotypic and genotypic changes and therefore preserves more characteristics of the original primary cell line are required.

Here we describe the development and preliminary characterisation of cell lines from a diverse range of tissues from *Pteropus alecto*, the black flying fox. This species was selected for a number of reasons: (i) there is evidence that this species is a reservoir host for Hendra virus; (ii) it is closely related to the flying-fox hosts of other bat-borne zoonotic viruses such as Nipah, Melaka and Kampar viruses; (iii) they have a natural distribution beyond Australia's shores into South East Asia; and (iv) there is an abundance of *P. alecto* colonies in South East Queensland.

## Results

### Comparison of Different Primary Cell Culture Methodologies

Initial trials comparing four different tissue culture methods (detailed in Materials and Methods) generated cell cultures of most tissue types with varying degrees of success. Generally, the methods using enzymatic digestion to break up the tissue (Methods 1 and 2) were more successful than the methods utilising physical disruption. (Methods 3 and 4). Method 2, trypsin treatment at 4°C overnight, was found to be the most effective and reliable in generating viable cell cultures across the majority of different tissue types. The comparatively long incubation time in trypsin allowed greater penetration and better digestion of the tissue as compared to Method 1, where trypsin treatment was at 37°C. The simplicity of Method 2 and its reproducibility led to the adoption of this method for our primary cell culture production. Contaminant-free cell cultures from intestine and skin were difficult to establish because of the obvious difficulty in obtaining tissues free from bacterial and fungal contamination.

Cell culture media was evaluated across the range of tissue type for optimal growth. Attempts to establish cell culture from tissues grown in Xten GO serum free medium was the least successful. The most successful cell culture medium across the majority of tissue types was found to be DMEM/F12-Hams. Supplementing media with bat serum as opposed to bovine calf serum appeared to make little difference to cell growth and so bovine calf serum was used for reasons of economy and convenience.

### Preliminary Characterisation of Primary Cell Lines

**Figure 1:**
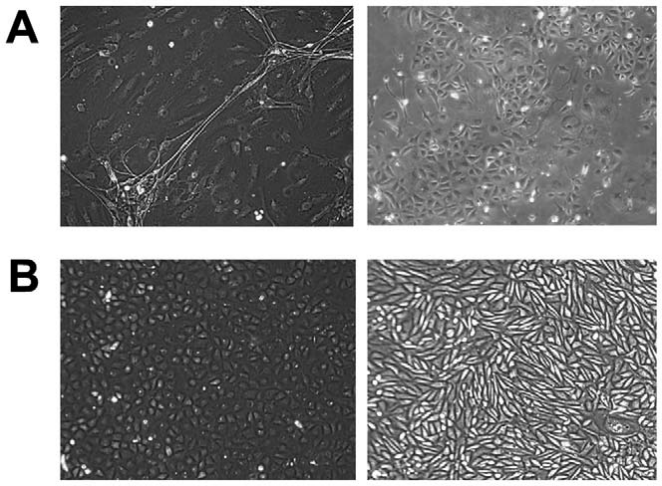
Morphological differences observed for primary cell cultures derived from *P. alecto* tissues. (A) Cells derived from a brain (left) and kidney (right) after 5 days in primary cell culture. (B) Cells derived from liver (left) and kidney (right) after 12 days in primary cell culture.

During the establishment of the primary cell cultures, non-adherent cells were lost during changes of medium. Only cells that attached to the culture flask were maintained and propagated by passage. The initial primary cell cultures derived from most tissues were heterogeneous, with a variety of cell morphologies observable (**Figure 1A**). The growth rate of primary cell cultures varied considerably, with cells from the aorta, kidney and foetus growing to confluence quickly and requiring passage within 6 days. By contrast, muscle, brain and lymph nodes took up to 15 days to reach confluence. Varying cell morphologies were observed, ranging from predominantly fibroblastic-like cells observed in the majority of tissues to cuboidal cells in cultures generated from lung and kidney (**Figure 1B**). Neural cells with dendrites were observed in cultures generated from brain.

Once the primary cell lines were established, cells from all tissue types (Table 1) grew well and were able to be passaged a number of times. As the cell cultures were passaged further, the monolayers became more homogeneous in appearance and the variety of cell types in each culture decreased (**Figure 1B**). Typical of nearly all primary cell cultures, the growth of non-immortalised primary cell cultures diminished significantly for most tissue types after approximately 10 passages.

**Table 1:** List of organs used for this study and status of immortalisation and cloning*.

Organ Type	Abbre-viation	Primary cell line established	Immortalisation and cloning			
			SV40T	Clone	hTERT	Clone
Aorta	Ao	Yes	PaAoT	ND	PaAoH	ND
Bone Marrow	Bm	Yes	PaBmT	ND	NS	ND
Brain	Br	Yes	PaBrT	PaBrT01-03	PaBrH	PaBrH01-07
Foetus	Fe	Yes	PaFeT	PaFeT01-10	PaFeH	NS
Foetal membranes	Fm	Yes	PaFmT	ND	NS	ND
Heart	He	Yes	PaHeT	ND	PaHeH	ND
Kidney	Ki	Yes	PaKiT	PaKiT01-03	PaKiH	NS
Liver	Li	Yes	PaLiT	ND	PaLiH	ND
Lymph Nodes	Ln	Yes	ND	ND	ND	ND
Lung	Lu	Yes	PaLuT	PaLuT01-04	PaLuH	NS
Muscle	Mu	Yes	PaMuT	ND	PaMuH	ND
Pharynx	Ph	Yes	ND	ND	ND	ND
Placenta	Pl	Yes	PaPlT	ND	PaPlH	ND
Salivary Gland	Sg	Yes	ND	ND	ND	ND
Small Intestine	Si	Yes	PaSiT	ND	PaSiH	ND
Skin	Sk	Yes	ND	ND	ND	ND
Spleen	Sp	Yes	PaSpT	ND	PaSpH	ND
Testes	Te	Yes	PaTeT	ND	NS	ND
Thymus	Th	Yes	ND	ND	ND	ND
Uterus	Ut	Yes	PaUtT	ND	PaUtH	ND

*Abbreviations: ND, not done; NS, not successful. Cell line nomenclature: first two letters indicated species (Pa  =  *Pteropus alecto*); second two letters represent the abbreviation of the original organ type from which the cell line was derived (e.g., Ki  =  kidney); the fifth letter indicates methods of immortalisation (T  =  SV40T and H  =  hTERT); the clone number is provided at the end in two-digit format (e.g., PaKiT01  =  clone #1 of the P. alecto kidney cells immortalised using the SV40T approach).

The identity of the bat cell lines established in this study was confirmed by two independent methods described in the Methods section. G-banding karyotyping demonstrated that the male *P. alecto* used for cell line development had 19 pairs of chromosomes, 18 pairs of autosomes plus one X and one Y (data not shown), with a similar morphology as that previously reported for a female *P. alecto* using R-banding [26]. A Pteropus-specific PCR was developed and validated using DNA extracted from the spleen of one female and one male *P. alecto* as well as DNA extracted from HeLa (human cervical cancer cell line), MDCK (Madin-Darby canine kidney), PK15 (pig), Vero (African Green monkey kidney), CHO (Chinese Hamster Ovary) and mouse heart tissue. While the predicted 454-bp fragment was obtained for *P. alecto* DNA, no PCR product was produced from the DNA samples of any of the other five mammalian species. Furthermore, sequencing of the *P. alecto* PCR product confirmed that it is highly conserved with the same region in the closely related *P. vampyrus* genome (data not shown).

### Immortalisation and Cloning

**Figure 2:**
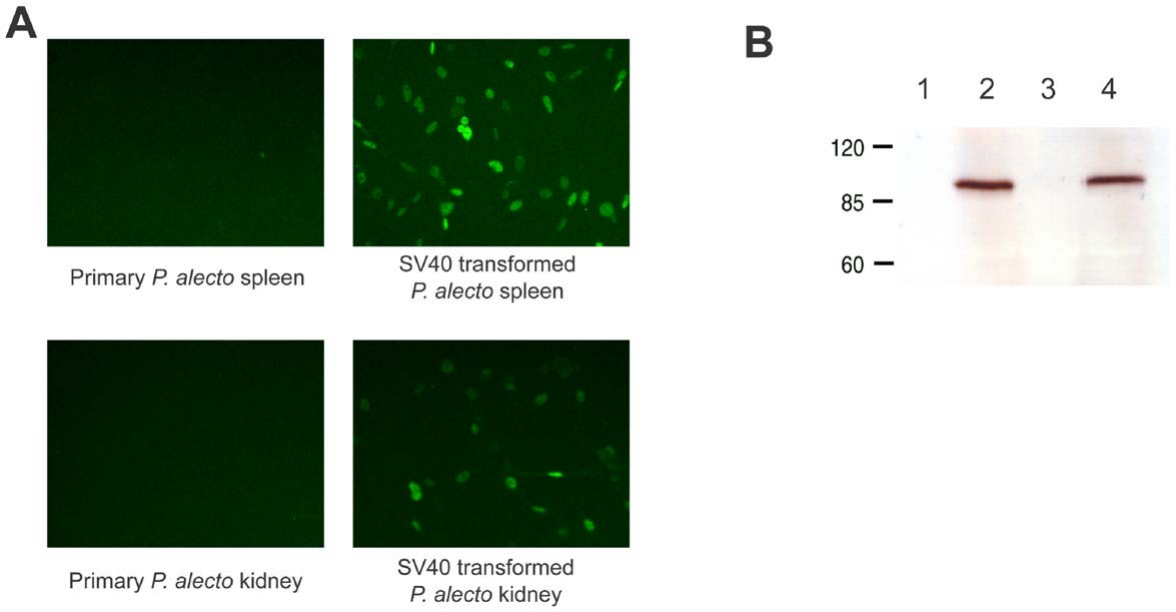
Stable expression of SV40T proteins in transformed cells. (A) Immunofluorescent staining of *P. alecto* spleen and kidney primary cells and cells transformed to detect expression of the SV40T antigens. (B) Western blot of untransformed *P. alecto* spleen and kidney primary cells (lanes 1 and 3, respectively) and transformed cells (lanes 2 and 4) to detect expression of the SV40 large T antigen.

Unlike rodent cells which are genetically relatively unstable [14], none of the bat primary cell lines established in this study appeared to have immortalised spontaneously. Therefore, two directed immortalisation strategies, i.e., the intracellular expression of SV40T or hTERT, were employed to transform the bat primary cell lines developed in this study. Both the SV40T and hTERT genes were introduced into bat cells using a retroviral vector system, which results in the stable integration of the introduced genes into the cellular chromosomal DNA [27]. Transformed cell lines are selected by using the hygromycin resistance marker encoded by the vector DNA. Expression of the SV40 large T antigen was confirmed by immunofluorescent antibody staining and Western blot, respectively (Figure 2). The expression of hTERT in transformed cells was also confirmed using the same methods (data not shown). Fifteen out of 20 primary cell lines were immortalised using the SV40 large T antigen approach and 12 using the hTERT approach (Table 1).

Cloning of the newly immortalised cells was considered an essential step in the establishment of the cell lines. Cloning will necessarily reduce the heterogeneity of the cell types present in the cell line. If performed optimally, cloning will ensure that the cell line is derived from a single cell type. This is critical to the production of cell lines that have consistent, reproducible characteristics. We were able to isolate single cells and grow viable cultures from those cloned cells. At the time of writing, five cloned, immortalised cell lines have been established (Table 1). The *Pteropus* origin of all the clones has been confirmed by the *Pteropus*-specific PCR (described above). Stocks from all cloned cell lines have been frozen in liquid nitrogen and then subsequently resurrected. Only SV40T or hTERT treated cells were able to be cloned and passed more than 10 times, providing additional evidence of successful immortalisation.

### Susceptibility to Infection by Nipah and Hendra Viruses

**Figure 3:**
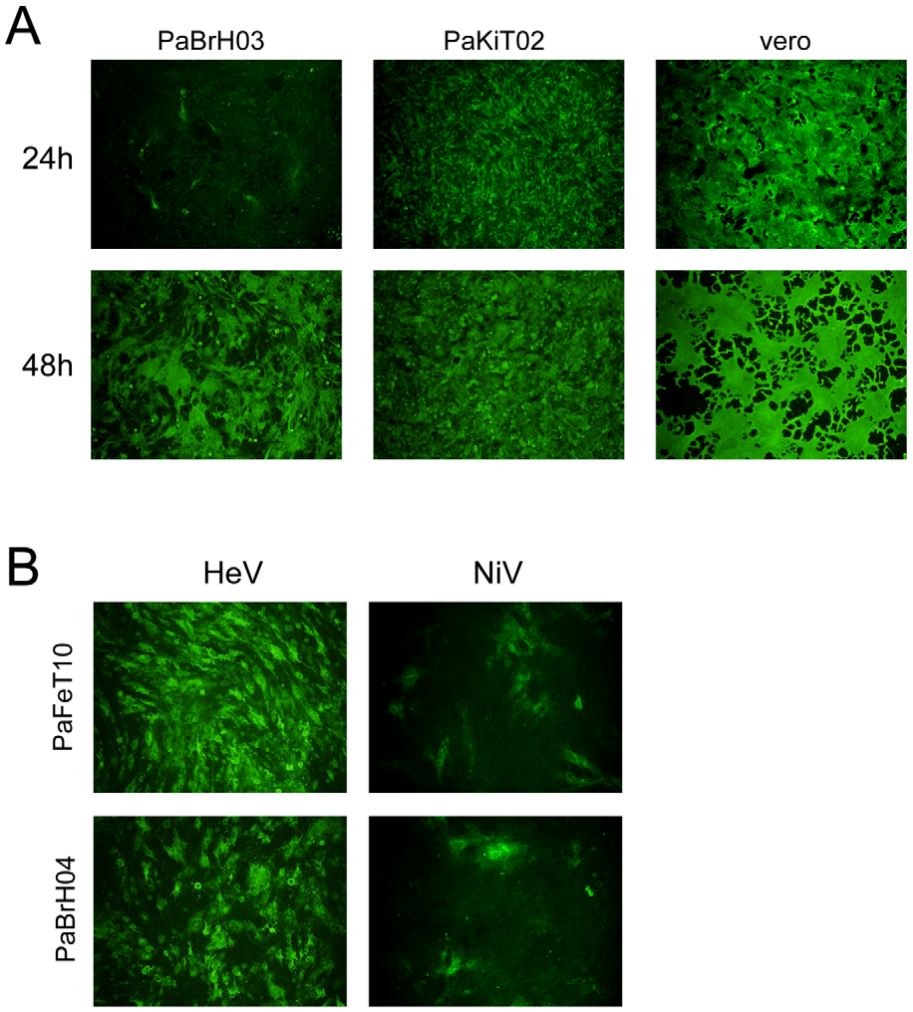
Infection of cloned *P. alecto* cell lines by HeV and NiV. (A) Comparison of infection kinetics of NiV in three different cell lines at 24 and 48 hours post infection. (B) Comparison of infection efficiency of *P. alecto* cloned cell lines for HeV and NiV. The images were taken 24 hours post infection. In both studies, cells were infected at high multiplicity of infection (MOI ≥100), fixed with 100% methanol and removed from the Biosafety Level-4 laboratory before being stained with HeV G protein-specific antibodies.

Variation in infectivity and viral protein production following high multiplicity infection with HeV or NiV was observed in the different primary cell lines. Although all primary *P. alecto* cell lines were successfully infected with both NiV and HeV, the infection efficiency was generally lower than that seen in the control infection in Vero cells (data not shown). In some primary cell cultures, only a small proportion of the cells produced a detectable level of viral protein expression 24 hours post infection (as detected by fluorescence, data not shwon). However, after 48 to 72 hours, all cell lines were producing greater quantities of viral proteins and for most primary cell lines every cell was infected. Only the primary heart cell line showed limited infection even after 72 hours (data not shown). The significance of this apparent difference is unclear. Similarly, in cloned immortalised cell lines, a difference was observed in infection kinetics in different lines. For example, NiV infected PaKiT02 cells (**Figure 3A**) produced detectable viral antigen levels comparable to that observed in Vero cells in almost all cells at 24 hours post infection, whereas the same infection in the PaKiH01 cells resulted in less than 25% of the cells producing detectable viral antigens at the same time point. However, at 48 hours, differences could no longer be seen.

In general, there was no observable difference between HeV and NiV infectivity in any of the primary cell lines. However, distinctive difference in infection efficiency was observed in some cloned immortalised cells, with HeV having higher infection efficiency. As shown in **Figure 3B**, HeV appears to have a much higher infectivity than NiV in the foetus (PaFeT10) and brain (PaBrH04) clones immortalised with SV40T and hTERT, respectively.

### Induction of Innate Immune Responses in Cloned Cell Lines

**Figure 4:**
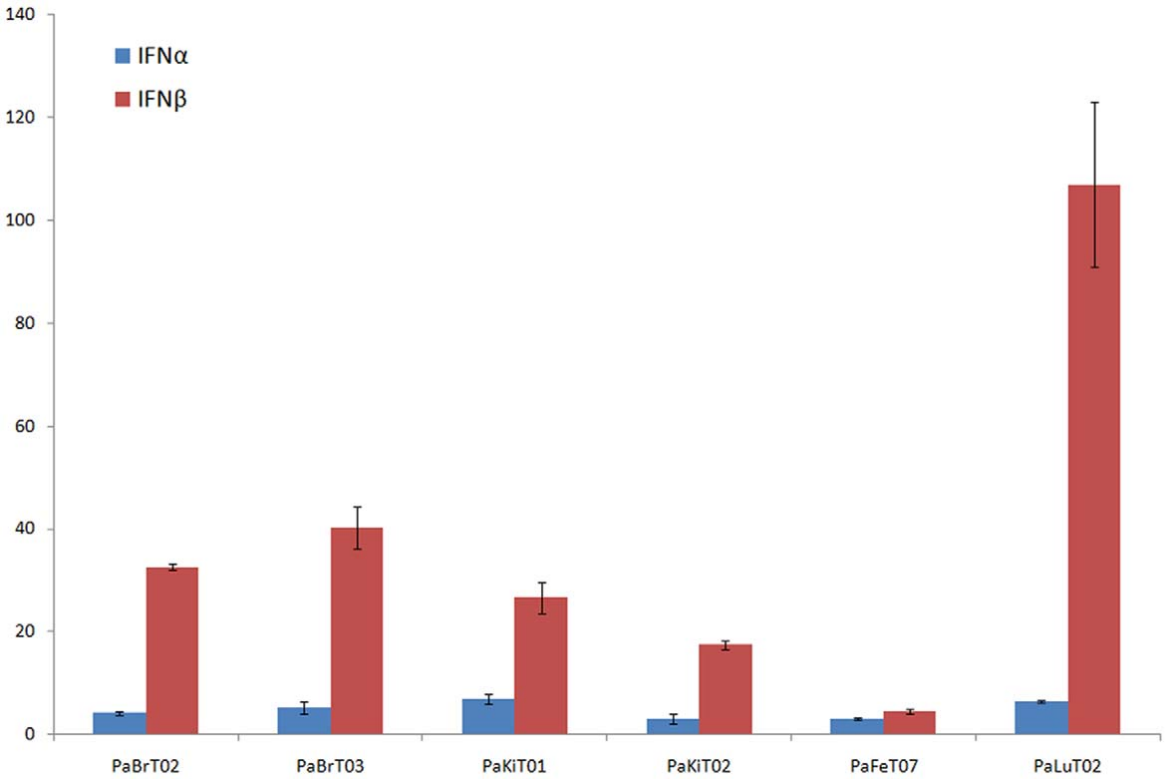
Induction of *P. alecto* interferon gene expression by poly I∶C. Results shown are of fold increase in IFN-α and IFN-β transcript levels (measured by real-time PCR) after treatment of *P. alecto* cloned and SV40 Large T antigen immortalised brain, kidney and foetal cells with 10 µg/ml poly I∶C over the basal level of IFN gene expression in mock treated cells. Error bars represent the standard deviation of the mean derived from duplicate samples.

One of the major applications of the cell lines established in this study will be for the investigation of the innate immune responses to infection by viruses of both bat and non-bat origin. As a first step towards the characterisation of the innate immune competency of different *P. alecto* cell lines, the stimulation of type I interferon gene expression by poly I∶C was examined in selected SV40T cloned immortalised cell lines. The results presented in **Figure 4** suggest that there is significant variation in the increase of type I interferon gene expression after poly I∶C treatment, from less than 10-fold increase in the PaFeT07 cells to more than 100-fold increase in the PaLuT02 cells. It is also interesting to note that the increase in IFN-β is greater than that for IFN-α in all the cell lines tested so far.

## Discussion

With the increasing trend of bat borne viruses crossing the species barrier and causing severe disease in humans and other animals, there is an urgent need for the establishment of cell lines from various bat species to facilitate virus isolation and basic research into virus-host interaction. This is especially important for the study of bat immune responses and their role in maintaining the symbiotic presence of a large number of viruses in bats, apparently without causing clinical disease.

In this study, we have chosen the Australian fruit bat, *Pteropus alecto*, as a model system to compare and select the best tissue culture and immortalisation methodologies for the establishment of stable bat cell lines. From a total of 20 different organs, we were successful in establishing primary cell lines for all and have generated immortalised clones from a selected group of cells originating from different organs (summarised in Table 1).

Of the two different disruption methods compared, the enzymatic treatment approach was superior to the physical disruption method. It was evident that the overnight treatment with trypsin at 4°C provided the best results, probably due to the better penetration of the enzyme solution. In comparison with treatment at 37°C for a fixed time, the overnight incubation at 4°C not only provided more flexibility when handling a large number of tissue culture samples at one time, it also generated more successful and reproducible results.

While both the SV40 Large T antigen and hTERT have been used to immortalise cells of human and other mammals [16], [17], [21], [23], [25], to our knowledge this is the first successful application of these approaches to cells of bat origin. The ability of hTERT to functional in another mammalian species and thus immortalise bat cells is not totally unexpected considering that hTERT is highly conserved across many eukaryotic species including the conservation of functional domains in lower eukaryotes [28]. Our study adds bats to the list of non-human mammalian species (pig [23], goat [20], cow [21] and dog [22]) for which hTERT can been used to produce immortalised cells. In general, there appears to be a more visible morphological change after immortalisation with SV40T in comparison to those treated with hTERT. This is not surprising since immortalisation with the SV40 oncogene typically results in genetic and phenotypic changes [16] whereas ectopic expression of hTERT results in the maintenance (or close to) of the original characteristics of the primary cell line it was derived from, including minimal karyotypic changes and non-malignant phenotype [22], [25], [29]. Despite the differences in the immortalisation methods, preliminary infection and innate immune response studies revealed no differences between the cell lines attributable to the immortalisation method. Further characterisation is required to determine if there are any important phenotypic differences.

Difference in infectivity and kinetics of virus replication was observed among both primary cells and cloned immortalised cells. It is interesting to note that among several cloned cell lines, HeV appears to have better infection efficiency than NiV. This was not totally unexpected considering that *P. alecto* is the natural reservoir for HeV in Australia whereas NiV is mainly found in *P. vampyrus* in Malaysia. Notably, the target receptor for NiV and HeV (ephrin B2 and B3) is almost identical in the two different bat species [30], making it likely that other cellular factors are responsible for the observed infectivity difference for by HeV and NiV.

Equally interesting is the observation that NiV displayed different infection kinetics in different cloned cell lines. At 24 hours post infection, there was at least 4–5 fold difference in the staining of viral antigens between PaBrH03 and PaKiT02. However, this difference was no longer visible at 48 hours post infection. This suggests that the NiV was able to enter and infect both cell types with almost 100% efficiency, but the rate of virus replication, measured by the production of viral antigens, varied significantly between the two lines. This also indicates that cellular factors, other than receptor molecules, might be responsible for influencing virus replication kinetics.

One explanation for the variation in virus replication may involve factors (viral and or cellular) involved in the regulation of innate immunity in *P. alecto* cell lines. Since the tools for examining bat innate immunity are very limited, in this preliminary study we have assessed innate immunity by measuring interferon alpha and interferon beta gene expression with and without the treatment of poly I∶C, a commonly used stimulator of interferon gene expression [31], [32]. As found in other mammalian species, a wide response range was observed between the bat cell lines tested. The correlation of interferon expression with infectivity and replication kinetics for different viruses is yet to be determined. This does, however, provide a useful tool for screening cell lines in the future to assess their suitability for the various research needs required by different studies. In parallel, we are producing more *P. alecto* specific reagents (real time PCR and antibodies) for a more comprehensive examination of innate immunity in different cell lines generated in this study.

In conclusion, we have established an optimised approach for generating stable immortalised cell lines from *P. alecto* bats. These cell lines are pivotal tools for our future studies of virus-bat interaction and the role of the bat immune system in controlling virus infection. Our preliminary infection and innate immune response studies confirmed the variation expected among different cells lines and the need to establish multiple and different cell lines to suit diverse research needs. We believe that the optimised method for generating stable immortalised cell lines developed in this study will be generally applicable to generation of cell lines from other bat species. To this end, we are in the process of generating cell lines from at least three additional bat species, including two microbat species.

## Materials and Methods

### Animals

*P. alecto* bats were captured in Brisbane, Queensland, at dawn using a mist net. They were transferred to a clean cotton pillow case, which was suspended inside a pet transport pack, for same day transportation by air to the Australian Animal Health Laboratory (AAHL) in Geelong. Bats were housed overnight at AAHL before being anaesthetised using a mix of ketamine (Parnell Laboratories) 3 mg/kg and medetomidine (Pfizer) 60 µg/kg delivered intramuscularly. Once anaesthetised, they were heart bled until exsanguinated. Tissues were removed from the bats and pooled into an appropriate volume of processing medium, depending on the size of the tissue, in specimen jars on wet ice. Processing medium consisted of magnesium- and calcium-free phosphate buffered saline (PBS) containing 200 mg/l disodium EDTA (Gibco), 100 units/ml Penicillin (CSL Ltd.) and 100 µg/ml Streptomycin (Sigma). Organs collected in this study are summarised in Table 1. In addition to the common organs taken from both male and female bats, testes were taken from a male bat and for one pregnant female bat, foetus, foetal membranes, placenta and uterus were taken.

### Ethics Statement

All animal work was conducted under conditions and with permits approved by animal ethics committees of the Australian Animal Health Laboratory and the Queensland Department of Primary Industries and Fisheries.

### Preparation of Primary Cell Cultures

A number of different methods were evaluated for the generation of primary cell cultures in order to determine the most appropriate conditions for bat cells. These included two variations of trypsinisation and two physical disruption techniques; explant and mechanical mesh strainer. For all organs other than intestine and skin, the tissues were first cut finely using a scalpel, washed with cold processing media and then divided equally for each cell culture method.

A number of different cell culture media was trialled including Xten GO serum free medium (Thermo), RPMI (Sigma), BME with Earle's salts (SAFC Biosciences), M199 (Sigma) and DMEM/F12-Hams (Sigma), each supplemented with 15% bovine calf serum (BCS, Hyclone), 100 units/ml penicillin, 100 µg/ml streptomycin and 50 µg/ml gentamycin (Sigma). RPMI was also trialled using 5% pteropid bat serum replacing the BCS. The concentration of BCS in all media was reduced to 10% once cultures were established.

#### Method 1: Trypsin-37

Cold 0.25% trypsin in PBS containing 200 mg/l disodium EDTA was added to the prepared tissue and incubated at 37°C on a shaking platform. After 10 min, the supernatant was poured through a gauze mesh into a tube containing 10 ml BCS. More trypsin was added to the undigested tissue and the process repeated twice after additional 10 minute incubations. Trypsinised cells were then divided equally into an appropriate number of tubes depending on the number of media being trialled and pelleted by centrifugation at 800 x g for 5 min. The cell pellets were resuspended into appropriate media, transferred into tissue culture flasks (Corning) and incubated in a humidified incubator with 5% CO_2_ at 37°C.

#### Method 2: Trypsin-4

Cold 0.25% Trypsin in PBS containing 200 mg/l disodium EDTA was added to the prepared tissue and placed at 4°C overnight. The tubes containing tissue were then incubated at 37°C on a shaking platform for 1 h. The large pieces of tissue were allowed to settle and supernatant was poured through gauze mesh into a tube containing BCS. Cells were then divided equally into tubes and subsequently processed under the same conditions as in Method 1.

#### Method 3: Explant

Prepared tissues were cut into small pieces and divided into tissue culture flasks with appropriate media. The tissue pieces were incubated in a humidified incubator with 5% CO_2_ at 37°C and were allowed to settle and attach over a number of days.

#### Method 4: Mesh Strainer

Prepared tissues fragments were poured into a 100-µm nylon mesh strainer (BD Falcon) and a sterile syringe plunger used to push tissue through mesh. The mesh strainer was washed with cold Eagle's Minimal Essential Medium containing Earle's salts (EMEM) containing penicillin/streptomycin. Cells were then divided equally into tubes and subsequently processed under the same conditions as in Method 1.

#### Method 5: Intestine

Intestine was placed into a Petri dish and a 5-ml syringe with 16 gauge needle attached to thin sterile tubing was used to draw up cold PBS. The tubing was carefully inserted into one end of the intestine and the intestine was washed 5 times by pushing through 5 ml of cold PBS per wash. One end of the intestine was then clamped using artery forceps and the intestine filled with pre-warmed trypsin solution. The other end was clamped and the intestine was then incubated at 37°C for 15 min. The intestine was then agitated before removing the clamps and the cells suspension was decanted into a new tube. Cells were then divided equally into tubes and subsequently processed under the same conditions as in Method 1.

#### Method 6: Skin

Skin was washed thoroughly with cold PBS and then cells were scraped off into cold PBS. Cells were then divided equally into tubes and subsequently processed under the same conditions as in Method 1.

In all cases, cell medium was removed after 3–5 days and replaced with fresh medium supplemented with 10% BCS.

### Preparation of Frozen Cell Stocks

Cell medium was decanted from confluent 150-cm^2^ tissue culture flasks and cells were washed once with PBS. Cold 0.25% trypsin in PBS was added to flasks and incubated for 2–10 min at 37°C. Cell medium containing 10% BCS was added to cells to inactivate trypsin and the cells pelleted at 400 x *g* for 2 min. The medium was decanted and the cell pellet resuspended in freezing medium consisting of cell media containing 20% BCS and 10% DMSO (Sigma). The vials were frozen at −80°C in cell culture freezing containers before being removed into vapour phase liquid nitrogen cabinets for long term storage.

### Characterisation of Bat Cells by Karyotyping and PCR

Primary kidney cells from a male *P. alecto* were grown to 70–80% confluency in DMEM/F12-Hams with 10% BCS, 100 units/ml of penicillin, 100 µg/ml of streptomycin and 1.25 µg/ml of amphotericin B (Sigma). A flask containing cells at passage 4 was filled with medium, sealed with Parafilm and sent to a commercial provider (TissuPath Pty Ltd, Melbourne, Australia) for karyotyping using g-banding (Giemsa stain). To confirm the identity of newly established *P. alecto* cell lines and to eliminate the possibility of contamination from cells of other species, a *Pteropus*-specific PCR was developed based on a large exon of the human Apolipoprotein B 100 Precursor gene (Ensemble Genome Browser ID #: ENSG00000084674). The nucleotide sequence of this exon from *Homo sapiens* was aligned (using the Ensembl genome browser) with the sequence from a microbat (*Myotis lucifugus*) and a fruit bat (*Pteropus vampyrus*) respectively as well from the following mammalian species: *Pan troglodytes, Macaca mulatta, Cavia porcellus, Mus musculus, Rattus norvegicus, Bos taurus, Canis familiaris, Equus caballus and Choloepus hoffmanni*. From the alignment, a *Pteropu*s-specific PCR primer pair (ApoB 3F, 5′ GGAGA AGCCA CTCTC CGACG 3′ and ApoB 5R, 5′ TAAGA TACTG TTTCC TCTCA GTAC 3′) was designed which is predicted to be specific for *Pteropus*, resulting in a 454-bp PCR product. PCR reactions were set up in a total volume of 25 µl with 12.5 µl 2x GoTaq Hot Start Green Master Mix (Promega), 100 ng of genomic DNA and 0.5 µM final concentration of each primer (ApoB 3F and ApoB 5R). The PCR cycling conditions were as follows: 2 min at 95°C then 40 cycles of 95°C for 30 sec, 64°C for 1 min and 72°C for 1 min with a final extension of 72°C for 5 min.

### Preparation of Retrovirus Vectors for Stable Expression of Foreign Genes in Bat Cells

In order to transform the *P. alecto* cells, genes coding for the SV40 small and large T antigen (SV40T) and the human telomerase reverse transcriptase (hTERT) were stably introduced into bat cells using a retrovirus transduction system. The SV40T and hTERT genes were cloned into pQCXIH (Clontech) and the resulting plasmid packaged into retrovirus particles in the GP2–293 packaging cell line (Clontech) and pseudotyped with vesicular stomatitis virus G glycoprotein (VSV-G) following the manufacturer's instructions.

### Retrovirus Transduction of Bat Cells for Transformation

Primary cell lines of low passage number (2–3 passages) were infected with the VSV-G pseudotyped retrovirus particles in the presence of 1 µg/ml polybrene (Sigma). Eight hours post infection, the medium was changed and the cells were allowed to recover, allowing time for the retroviral insert to be incorporated into the bat cell genome and for expression of the hygromycin resistance gene product. Forty eight hours post infection, cells transformed by the retrovirus were selected for by the addition of 10 µg/ml hygromycin in the media. Stocks of cells that were resistant to hygromycin were prepared and frozen. Expression of SV40 Large T antigen or hTERT was monitored by immunofluorescent antibody assay (IFAT) and Western blot analysis. For IFAT, cells were fixed with methanol and stained with antigen-specific mouse antibodies, mouse anti-SV40T monoclonal antibody (Abcam, Cat# ab16879) and mouse anti-hTERT polyclonal antibody (Abcam, Cat# ab52810), followed by goat anti-mouse FITC (Chemicon). For Western blot, the same mouse antibodies were used as primary antibody and bound antibodies were detected with goat anti-mouse alkaline phosphatase (MP Biomedicals). Reactive signals were visualised using the BCIP/NBT Color Development Substrate (Promega).

### Cloning of Transformed Cells

Following transformation, cells were trypsinised and diluted in cell culture medium to give a concentration of one cell per 100 µl. This suspension was aliquoted into 96-well tissue culture plates to give approximately one cell/well. The plates were incubated at 37°C in a humidified 5% CO_2_ incubator. Each well was monitored for cell growth and confluency. Once a well became confluent, the cells were trypsinised and passaged into a well of a 24-well tissue culture plate. Once cells had grown to 90-100% confluency they were passaged into a 25-cm^2^ tissue culture flask and continued to be passaged into larger flasks until sufficient numbers were generated for storage.

### Preparation of Hendra and Nipah Virus Stocks

Two isolates of Hendra virus, Hendra (HeV-H) and Redlands (HeV-R), and two isolates of Nipah virus, Malaysia (NiV-M) and Bangladesh (NiV-B), were used to infect *P. alecto* cell lines. All viruses were grown in Vero cells in EMEM supplemented with 10% BCS, 100 units/ml of penicillin and 100 µg/ml of streptomycin. The viruses were passaged at a multiplicity of 0.05 TCID_50_/cell to generate working stocks of each. Both HeV-H and HeV-R grew to titres of 1×10^8^ TCID_50_/ml, while NiV-Malysia had a titre of 2×10^8^ TCID_50_/ml and NiV-Bangladesh had a titre of 7×10^6^ TCID_50_/ml.

### Virus Infection and Analysis by Immunofluorescence Microscopy

Wells of 96-well tissue culture microtitre plates were seeded with each of the *P. alecto* cell culture lines at 2×10^4^ cells/well in 100 µl medium containing 10% BCS and incubated at 37°C in a humidified incubator with 5% CO_2_ for 24 h. Vero cells were used as a positive control and seeded under the same conditions. Plates were transferred into the Biosafety level-4 laboratory where they were infected with each virus isolate at a multiplicity of infection above 100. The plates were incubated at 37°C in a humidified 5% CO_2_ incubator. Following incubation for 24, 48 or 72 h, the culture medium was discarded and the plates immersed in absolute methanol for 10 min to fix and inactivate virus prior to removing from the PC4 laboratory. Plates were allowed to air dry before blocking with 100 µl/well of 1% BSA in PBS and incubating at 37°C in a humidified 5% CO_2_ incubator for 30 min. Buffer was discarded and polyclonal rabbit sera raised against HeV sG protein was diluted 1∶100 in blocking solution, 50 µl added to each well and incubated at 37°C in a humidified 5% CO_2_ incubator for 30 min. Plates were washed 3 times with PBS containing 0.05% v/v Tween 20 (PBS-T) before addition of 50 µl/well Alexa Fluor 488 goat anti-rabbit fluorescent conjugate (Molecular Probes) diluted 1∶1000 in blocking solution and incubated at 37°C in a humidified 5% CO_2_ incubator for 30 min. Plates were washed 3 times with PBS-T and 100 µl of PBS was added to each well before viewing with an Olympus IX71 fluorescent microscope.

### Induction of Interferon Responses in Cloned Cell Lines

Confluent cell monolayers of cloned *P. alecto* cells were transfected with 10 mg poly I∶C (Sigma) using 2 µl of Lipofectamine 2000 (Invitrogen) following manufacturer's instructions. Three hours post transfection, cells were harvested and RNA extracted using a RNeasy minikit (Qiagen). RNA was reverse transcribed using random primers and Superscript III (Invitrogen) and quantitative PCR performed on an Applied Biosystems 7500 Fast Real-Time PCR System and EXPRESS SYBR® GreenER™ qPCR Supermix Universal (Invitrogen) using the following PCR conditions: 95°C for 3 s followed by 40 cycles of 95°C for 3 s and 60°C for 30 s. Primers for amplification of GAPDH, interferon α and interferon β were designed based on the *P. vampyrus* genome sequence available on NCBI. Primer sequences are given in Table 2. Quantification was achieved by normalisation of GAPDH and expressed as fold increase compared to mock treated cells.

**Table 2:** Primers used for quantitative PCR analysis of gene transcription in cloned *P. alecto* cells.

Primer name	Primer sequence
GAPDH forward	ATACTTCTCATGGTTCACAC
GAPDH reverse	TCATTGACCTCAACTACATG
Interferon α forward	TGAGATCCTGCTAGGCAGGTTC
Interferon α reverse	GGCACAAGGGCTGTGTTTCTTC
Interferon β forward	TTCATTCCAGCCAGTGCTAGAG
Interferon β reverse	TCCCTGCGGAGATTAAACAACC
